# Identification of two proteins that interact with the Erp virulence factor from *Mycobacterium tuberculosis *by using the bacterial two-hybrid system

**DOI:** 10.1186/1471-2199-10-3

**Published:** 2009-01-21

**Authors:** Laura I Klepp, Marcelo Soria, Federico C Blanco, María V Bianco, María P Santangelo, Angel A Cataldi, Fabiana Bigi

**Affiliations:** 1 Institute of Biotechnology, CICVyA-INTA Castelar, Nicolas Repetto and Los Reseros, 1686, Hurlingham, Argentina; 2 Microbiología Agrícola Facultad de Agronomía. Universidad de Buenos Aires, Av. San Martin 4453, 1417 Buenos Aires, Argentina

## Abstract

**Background:**

The exported repetitive protein (*erp*) gene encodes a secreted 36-kDa protein with a central domain containing several proline-glycine-leucine-threonine-serine (PGLTS) repeats. It has been demonstrated that *erp *is a virulence-associated factor since the disruption of this gene impairs the growth of *Mycobacterium bovis *and *Mycobacterium tuberculosis *in mice.

**Results:**

In order to elucidate the function of Erp we searched for Erp-binding proteins from *M. tuberculosis *by using a bacterial two-hybrid system. Our results indicate that Erp interacts specifically with two putative membrane proteins, Rv1417 and Rv2617c. Further analysis revealed that the latter two interact with each other, indicating that Rv1417, Rv2617c and Erp are connected through multiple interactions. While Rv1417 is disseminated in several *Actinomycetales *genera, orthologues of Rv2617c are exclusively present in members of the *M. tuberculosis *complex (MTC). The central and amino-terminal regions of Erp were determined to be involved in the interaction with Rv1417 and Rv2627c. Erp forms from *Mycobacterium smegmatis *and *Mycobacterium leprae *were not able to interact with Rv2617c in two-hybrid assays. Immunolocalization experiments showed that Rv1417 and Rv2617c are found on the cell membrane and Erp on the bacterial cell wall. Finally, comparative genomics and expression studies revealed a possible role of Rv1417 in riboflavin metabolism.

**Conclusion:**

We identified interactive partners of Erp, an *M. tuberculosis *protein involved in virulence, which will be the focus of future investigation to decipher the function of the Erp family protein.

## Background

*M. tuberculosis *Erp (Rv3810) and *M. bovis *P36 (Mb3840) are homologous 36 kDa proteins that comprise 284 amino acids (aa) and possess a classical signal sequence. The central segment has 11 PGLTS repeats, four of which match exactly with the consensus and seven are degenerate. The export signal sequence consists in four charged aa followed by 14 nonpolar ones and a probable cleavage site for the signal peptidase. Erp and P36 have been detected only in culture supernatants and cell wall preparations, but not in cell extracts [1-3].

De Mendoça et al. have demonstrated that orthologues of the *erp *gene are also present in saprophytic and environmental opportunistic pathogenic mycobacteria [4]. A striking feature of this family is that it has no orthologous sequences outside the *Mycobacterium *genus. Thus, it can be considered a *Mycobacterium*-specific family of secreted proteins.

Although the precise roles of Erp proteins have remained elusive, the number of reports showing that Erp is a crucial factor for survival and multiplication of bacteria both *in vitro *and in animal models is increasing. The initial evidence supporting a role of the Erp protein in mycobacterial pathogenesis came from a study by Berthet *et al*., who demonstrated that the disruption of *erp*/*p36 *in both *M. tuberculosis *and *M. bovis *BCG negatively affects the multiplication of these strains in infected cultured bone marrow-derived macrophages and mice [2]. In agreement with these results, disruption of *p36*, impairs the growth of virulent *M. bovis in vivo *[5]. Finally, it has been reported that *erp*-deficient *Mycobacterium marinum *has an attenuated growth in cultured macrophage monolayers and during chronic granulomatous infection of leopard frogs, its natural host species. These results suggest that the function of Erp is similarly required for the virulence of *Mycobacterium *species other than those belonging to the MTC [6]. It has also been shown that *erp*-deficient bacteria are attenuated primarily because of reduced intracellular growth and/or survival in macrophages from zebrafish embryos [6]. Thus, these findings reinforce the notion of Erp as a virulence factor of pathogenic mycobacteria. However, the exact function of this virulence factor during host infection is still unknown.

Because Erp has several central repeat regions, we hypothesized that these regions participate in the interaction with other proteins. In order to gain insights into the function of Erp, and based on the premise that the function of unknown proteins may be discovered through their interaction with a protein target with a known function, we searched for Erp-binding proteins from *M. tuberculosis *by using a bacterial two-hybrid system. We here report that Rv1417 and Rv2617c were able to interact with Erp and that these proteins relate to each other through multiple interactions. In addition, important aspects of the association of Erp with mycobacterial virulence are discussed.

## Results

### 1. The Erp protein interacts with Rv1417 and Rv2617c in a bacterial two-hybrid system

We used a two-hybrid system developed by Ladant and co-workers [7], in which genes of interest are fused to T18 and T25, two complementary fragments that are essential for adenylate cyclase activity. If the corresponding fusion proteins interact, cAMP is produced in an endogenous adenylate cyclase-deficient *E. coli *strain (BTH101), and this functional complementation can be easily monitored by plating bacteria in minimal medium supplemented with lactose. In this work, we searched for Erp-binding proteins by screening an *M. tuberculosis *DNA expression library with full-length Erp using this bacterial two-hybrid system. The size of the library was approximately 10^5 ^clones. Out of 6 × 10^3 ^plated transformants, 10 *cya*+ clones which could grow in minimal medium supplemented with lactose were selected, indicating ten potential interactions. Enzymatic restriction analysis revealed that clones were unique (data not shown). In order to confirm the interactions and to exclude "false positives", plasmids were purified and used to retransform *E. coli *BTH101. In this second round, only three plasmid clones whose products were able to confer adenylate cyclase activity in *E. coli *BTH101 co-transformed with plasmid T25-Erp were selected. Sequence analysis of inserts revealed that two of these plasmids encoded Rv1417 and one Rv2617c. The plasmids encoding Rv1417 had a complete copy of the gene and they differed only in the length of the 5' region upstream of *Rv1417*, while in the plasmid encoding Rv2617c the first 60 bp of the gene were absent. Rv1417 and Rv2617c were annotated as membrane proteins, both of unknown function [8]. All sequenced fragments were in-frame with the ORF encoding T18. The fact that both plasmids encoding Rv1417 were independent clones confirms that non-redundant clones were present in the genomic library and reinforces the feasibility of Erp-Rv1417 protein interaction.

**Figure 1:**
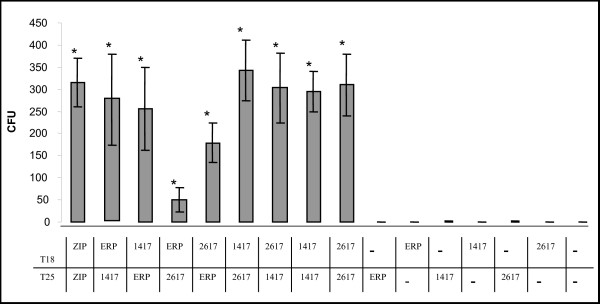
***In vivo *interaction of Erp, Rv1417 and Rv2617c**. *E. coli *BTH 101 cells were transformed with the plasmids described in the figure and plated in M63 medium supplemented with lactose. Colonies were counted after five days of culture. Triplicate plates were prepared. The bars represent the mean number of colonies ± S.D. *Significantly different (P < 0.05) from the value of negative controls as calculated by the Student's t test. ZIP: positive control, 1417: Rv1417, 2617: Rv2617c, ERP: Rv3810.

In order to examine the protein-protein interactions mentioned above, *erp*, *Rv1417 *and *Rv2617c *full-length genes were fused to both T18 and T25 gene sequences in pUT18c and pKT25 vectors, respectively. The efficiencies of functional complementation between hybrid proteins were determined by the number of colonies grown in M63 medium supplemented with lactose, and by β-galactosidase activity (see Additional file 1). The level of interaction between Erp and both Rv1417 and Rv2617c was significantly higher than that of the negative controls, independently of the adenylate cyclase fragments (T25 or T18) these proteins were fused to (Fig. 1). These interactions were confirmed by *in vitro *GST-Pull down assays (see Additional file 2).

### 2. Rv1417 and Rv2617c interact with each other

The binding of Erp to two different proteins raised the question whether these two proteins are able to interact with each other. In order to clarify this point, the binding between Rv1417 and Rv2617c was addressed by using the bacterial two-hybrid system. These experiments were facilitated by the availability of constructs with each gene in both vectors that had been prepared for the present work. Plasmids (encoding protein fusions of Rv1417 and Rv2617c with T25 and T18 polypeptides) were used to transform *E. coli *BTH101 cells. All plasmid combinations were subjected to a quantitative screening on selective medium plates. Each hybrid protein tested was able to associate with the other partners (Fig. 1 and Additional file 1). Indeed, both Rv1417 and Rv2617c exhibited strong self-associations, thus suggesting homodimer complex formation of these proteins (Fig. 1 and Additional file 3). The Erp fusions, however, were impaired in self-association (data not shown). None of the hybrid proteins gave a significant complementation signal when tested either with control T25 and T18 polypeptides or with unrelated proteins, like lipoprotein P27 [9].

### 3. The carboxy-terminal domain of Erp is not relevant for protein interactions

**Figure 2:**
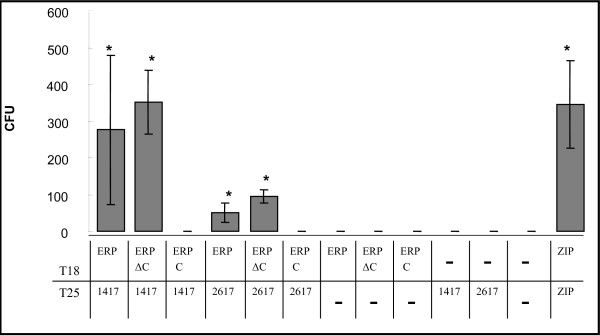
**Mapping of the Erp interacting domain**. The experiment was performed as in Fig. 1. ZIP: positive control, 1417: Rv1417, 2617: Rv2617c, ERP: Rv3810, ERP C: carboxi-terminal domain of Erp, ERP ΔC: Erp with its carboxy-terminal domain deleted.

In an effort to map the Erp region involved in protein-protein interactions, we assessed the capacity of each Erp domain to bind both Rv1417 and Rv2617c. Firstly, we focused on determining whether the carboxy-terminal region of Erp, which is involved in the association with the cell wall [10], contained a binding domain. The full sequence of the *erp *gene was divided in two regions at the base number 528, and fusions of both regions to the T18 encoding sequence were generated. The resulting hybrid proteins were then tested in two-hybrid complementation assays with Rv1417 and Rv2617c fused to the T25 fragment. Deletion of the carboxy-terminal region of Erp did not affect its association with Rv1417 and Rv2617c, thus suggesting that this region of the protein is not essential for this interaction (Fig. 2).

As a second step, the interacting region of Erp was further divided at aa 80, and fusion proteins of the amino-terminal and central domains of Erp with T18 polypeptide were generated. When separated, the amino-terminal and the central domains were unable to interact with Rv1417 and Rv2617c (data not shown). Only the protein deleted in the carboxy-terminal region showed a level of interaction comparable to, or even higher than, that of the full-length protein.

### 4. Interaction of Erp members from *Mycobacterium smegmatis *and *Mycobacterium leprae*

**Figure 3:**
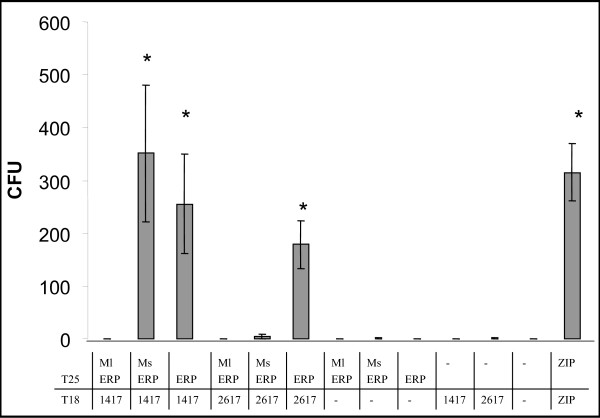
**Interaction of Erp members from *M. smegmatis *and *M. leprae *with Rv1417 and Rv2617c**. The experiment was performed as in Fig. 1. ZIP: positive control, 1417: Rv1417, 2617: Rv2617c, ERP: Rv3810. Ml ERP: *M. leprae *Erp homologue (ML0091), Ms ERP: *M. smegmatis *Erp homologue (MSMEG6405).

As mentioned in the introduction section of this work, Erp carboxy- and amino-terminal domains are fully conserved, while the central region shows polymoprhism among mycobacterial species, with respect to the number and quality of repeats. While *M. leprae *has four repeats, *M. smegmatis *has twenty-six, half of which contain two mismatches [4]. Therefore, it was plausible that the interaction with Rv1417 and Rv2617c was affected by the number of repeats. In order to evaluate this assumption, the interaction of *M. smegmatis *and *M. leprae *Erp homologues with both Rv1417 and Rv2617c was assayed. *M. leprae *Erp (Ml Erp) was unable to associate with either Rv1417 or Rv2617c. *M. smegmatis *Erp (Ms Erp) showed interaction with Rv1417 but completely failed to bind Rv2617c (Fig. 3). Although we can not exclude the possibility that the lack of interaction of Rv1417 and Rv2617c with the Erp member from *M. leprae *was due to a misfolding of the T25-Ml Erp protein, the impossibility of this fusion protein and of the T25-Ms Erp protein to interact with Rv2617c is interesting since it correlates with the absence of a functional *Rv2617c *gene in the *M. leprae *and *M. smegmatis *genomes (see below). On the other hand, the interaction of Rv1417 with the Erp member of *M. smegmatis*, but not with the one from *M. leprae*, indicates that the number and sequences of the PGLTS repeats are relevant for the occurrence of such interaction.

### 5. Erp, Rv1417 and Rv2617c are located in close proximity

**Figure 4:**
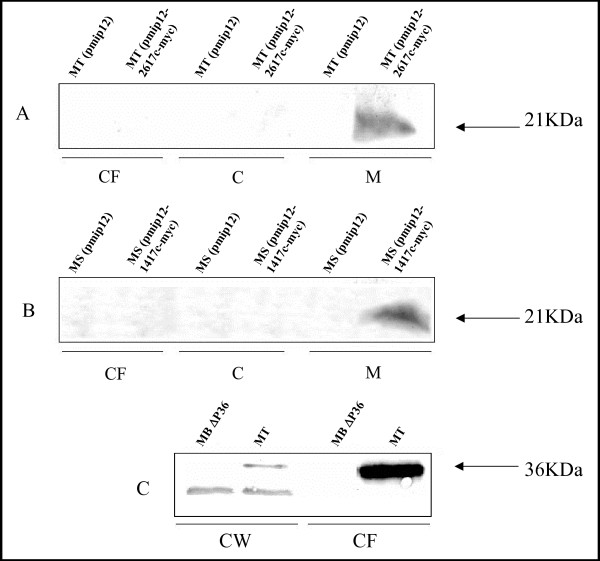
**Cellular localization of Rv1417, Rv2617c and Erp**. Myc-Rv1417 and Myc-Rv2617c were expressed in *M. smegmatis *and *M*. *tuberculosis*, respectively. Proteins from culture filtrates (CF), cytoplasm^©^, plasma membrane (M) and cell wall (CW) were extracted from the strains indicated above each lane and separated by 12% SDS-PAGE. Bands were detected either with anti-Myc Mab (Sigma-Aldrich) at a 1:100 dilution (A and B) or with anti-P36/Erp Mab [5] at 1:500 dilution^©^. MT: *M. tuberculosis*, MT (pmip12-2617c-myc): *M. tuberculosis *(pmip12-2617c-myc), MT (pmip12): *M. tuberculosis *(pmip12), MS (pmip12-1417-myc): *M*. *smegmatis *(pmip12-1417-myc), MS (pmip12): *M*. *smegmatis *(pmip12), MB P36: *M. bovis *ΔP36.

In order to determine the localization of the potential protein complex Erp-Rv1416-Rv2617c in mycobacterial cells, we first performed an *in silico *search for protein domains in Rv1417 and Rv2617c. Sequence analysis with InterProScan [11] recognized a signal-peptide domain in Rv1417 and Rv2617c. However, this was not confirmed by SOSUI or SignalP [12,13], two different software programs that perform signal peptide predictions. Both SOSUI and TMHMM [14] predicted the presence of two and three transmembrane helices for Rv1417 and Rv2617c, respectively. The analysis performed with the SOSUI server showed two transmembrane helices (encompassing positions: 22–44 and 51–72) in Rv1417 and three transmembrane helices (encompassing positions: 19–41, 81–103 and 117–139) in Rv2617c. TMHMM predicted that the probability of an extracellular location for the C-terminal region of Rv1417 was 0.36 and 0.64 for a cytosolic orientation. The predicted probability for an outward orientation for the C-terminus of Rv2617c was approximately 0.82. These results indicate a probable membrane localization of these proteins. To confirm these predictions, we performed immunolocalization of the proteins in subcellular compartments and in culture supernatants by using specific antibodies. Rv1417 and Rv2617c were expressed as a fusion to the myc epitope (see Materials and Methods) to allow their detection in mycobacterial cells. Since attempts to detect Rv1417-Myc in *M. tuberculosis *were unsuccessful, probably due to a very low expression of the recombinant protein, the fused protein was expressed in the *M. smegmatis *strain mc^2 ^155. Figure 4 shows the localization of Rv1417-Myc and Rv2617c-Myc in the membrane fraction of recombinant *M. smegmatis *and *M. tuberculosis*, respectively, but not in those transformed with the empty vector. Therefore, the co-localization of Rv1417 and Rv2617c suggests that protein-protein associations may take place in the cell envelope. In agreement with previous studies [1,2], Erp was identified in the cellular wall fraction and culture supernatant of *M. tuberculosis *by using a monoclonal specific antibody [5]. The absence of reacting bands in a *P36*-deficient *M. bovis *strain verified the antibody specificity.

### 6. Characterization of interacting proteins

In a BLAST [15] comparison of the predicted amino acid sequences of Erp interacting proteins, Rv1417 appeared to be conserved among the *Mycobacterium *genus and showed similarity to hypothetical membrane proteins from other bacterial species, such as *Rhodococcus sp *(identity 48%, similarity: 67%), *Corynebacterium ammoniagenes *(identity: 37%, similarity: 58%, with RibX protein), *Corynebacterium diphterae *(identity: 35%, similarity: 59%), *Streptomyces coelicolor *(identity: 34%, similarity: 55%) and *Streptomyces ambofaciens *(identity: 34%, similarity: 57%). Conversely, the *Rv2617c *gene was observed to be disseminated only among members of the *M. tuberculosis *complex (MTC). A pseudogene similar to *Rv2617c *was observed to be present in the *M. leprae *genome. The deduced amino acid sequence of *Rv2617c *showed similarity to hypothetical membrane proteins from *Rhodococcus sp *(identity: 48%, similarity: 67%), *Nocardoides sp *(identity: 61%, similarity: 72%), and *Arthrobacter sp *(identity: 56%, similarity: 72%).

**Figure 5:**
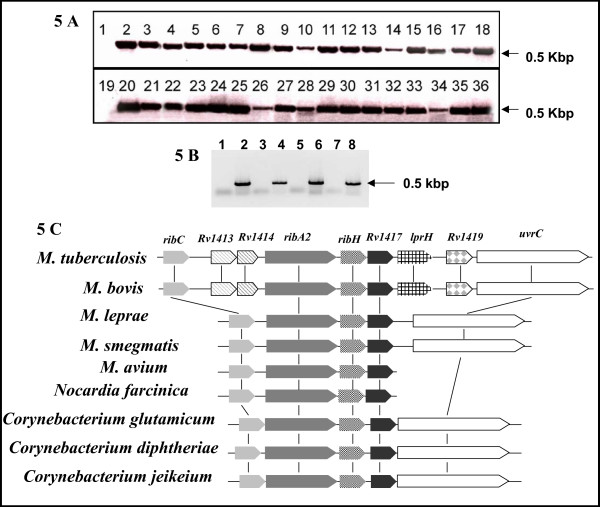
**Genetic characterization and expression studies of *Rv1417 *and *Rv2617c***. **A. Distribution of *Rv1417 *and *Rv2617c *in species of the MTC**. PCR amplifications of *Rv1417 *(lanes 1–18) and *Rv2617c *(lanes 19–36) were performed with pairs of primers 1417pktup/1417pktlow and 2617pktup/2617pktlow (table 1), respectively, and using the following genomic DNA as template: lanes 2 and 20, *M. tuberculosis *H37Rv; lanes 3–7 and 21–25, *M. microti *isolates; lanes 8–12 and 26–30, *M. pinnipedii *isolates; lanes 13–17 and 31–35, *M. bovis *isolates; lanes 18 and 36, *M. caprae *isolate. PCR negative controls were included (lanes 1 and 19). Arrows indicate the size of the bands. **B. Transcription of *Rv1417 *and *Rv2617c *in *M. tuberculosis***. The transcription of *Rv1417 *(lanes 1–4) and *Rv2617c *(lanes 5–8) was studied by RT-PCR assays using the pairs of primers 1417pktup/1417pktlow and 2617pktup/2617pktlow (table 1), respectively. Lanes 1 and 5, PCR negative controls; lanes 2 and 6, *M. tuberculosis *DNA (positive control); lanes 3 and 7, RT-PCR amplifications without RT; lanes 4 and 8, RT-PCR amplifications with RT. Arrow indicates the size of the bands. **C. Genomic organization of *Rv1417 *homologous *loci *in *Actinomycetales***. Schematic representation of genes encoding conserved proteins in the neighbourhood of *Rv1417 *in *Actinomycetales *genomes. Genes encoding homologous proteins are depicted in colours or patterns. Comparative genomic analysis was carried out with the STRING software and BLASTP analysis of the protein sequences deduced from genomic data bases.

In order to experimentally analyse the distribution of *Rv2617c *and *Rv1417 *in the MTC, PCR assays using specific primers were performed on genomic DNA from MTC species. DNA fragments of expected size were obtained for each gene in all species studied (Fig. 5A). In addition, the transcription of Rv1417 and Rv2617c during the *in vitro *culture of *M. tuberculosis *was demonstrated by RT-PCR (Fig. 5B). These results suggest that Rv1417 and Rv2617c are functional genes conserved in the MTC.

**Figure 6:**
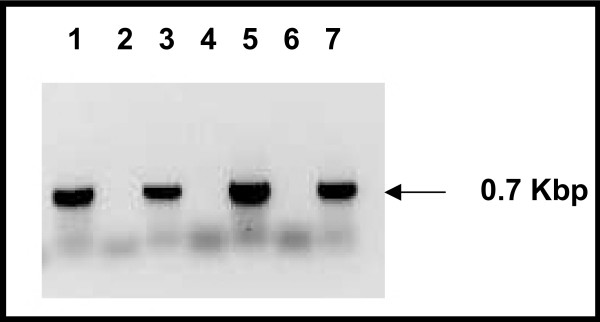
**RT-PCR analysis of *Rv1417 *and *RibH *in *M. tuberculosis *and *M. bovis***. Co-transcription of *Rv1417 *and *RibH *was studied by RT-PCR assays. Total RNA from *M. tuberculosis *H37Rv (lanes 1 and 2) and from *M. bovis *AN5 (lanes 3 and 4) was reverse-transcribed and amplified with the primers LeftOP1417/RightOP1417 (table 1). Lanes 1 and 3, RT-PCR amplification with RT; lanes 2 and 4, RT-PCR amplification without RT; lane 5, *M. tuberculosis *DNA (positive control); lane 6, PCR negative control; lane 7 *M. bovis *DNA (positive control). Arrow indicates the size of the bands.

We investigated the neighbourhoods of *Rv1417 *and *Rv2617c*, as well as of their orthologues, with the aim of obtaining clues regarding the biological role of these genes. While examination of the genomic location of the *Rv2617c *and its orthologues did not reveal any particular feature, we found that Rv1417 and its orthologues are upstream flanked by genes encoding for proteins involved in riboflavin synthesis. Riboflavin operons with a similar structure, containing an *Rv1417*-like gene, were identified in all *Mycobacterium *species whose genomes were sequenced, as well as in other species of *Actinomycetales *genera (Fig. 5C). In addition, RibX, whose gene is a putative member of the riboflavin operon of *C. ammoniagenes*, showed similarity to Rv1417 (E = e^-22^). Although the role of RibX in riboflavin synthesis remains elusive, a DNA fragment that includes part of the *ribX *gene was demonstrated to be involved in riboflavin production [16]. To determine whether *Rv1417 *is co-transcribed with *RibH*, a gene encoding a probable riboflavin synthase beta chain in *M. tuberculosis *and *M. bovis *strains, RT-PCR assays were performed by using primers that map across the two adjacent genes. The RT-PCR products shown in figure 6 indicate that *Rv1417 *and *RibH *are transcribed to a single mRNA molecule. Based on these results, we propose *Rv1417 *as part of the riboflavin operon in *M. tuberculosis*.

## Discussion

The availability of the *M. tuberculosis *genome sequence has provided us with new information, knowledge and understanding of the biology of this major pathogen as well as raised a number of questions concerning the roles and functions of a large group of putative unknown proteins, in which Erp/P36 is included. Here we present findings that could contribute to decipher the function of Erp in *M. tuberculosis*. The construction of a two-hybrid library expressing *M. tuberculosis *proteins enabled us to identify two Erp interacting proteins, Rv1417 and Rv2617c. We found that Rv1417 and Rv2617c are similar to hypothetical membrane proteins from other bacterial species but not to characterized proteins.

By BLAST searches, we identified Rv1417 orthologues in several mycobacterial species, including *M. smegmatis *and *M. leprae*. The presence of Rv2617c seems to be restricted to the *M. tuberculosis *complex, as no functional orthologues were identified in non-tuberculous species while *Rv2617c *genes were found by PCR in *Mycobacterium microti*, *Mycobacterium pinnipedii, Mycobacterium canetti *and *M. bovis *genomes.

By protein-protein interactions we also demonstrated association between Rv1417 and Rv2617c. Importantly, in all cases, complementation between the T25 and the T18 hybrids was detected in both configurations, that is, when the given proteins were fused to either the T25 or the T18 polypeptides. Moreover, both Rv1417 and Rv2617c, but not Erp, showed self-interaction, indicating that the former are able to generate homodimers. Taken together, these results show that it is likely that the three proteins form a heteromultimeric protein complex.

However, at this point of the investigation we do not know whether more than two units of Rv1417 and Rv2617c are assembled in the putative multi-protein complex.

Since the signal sequence of Erp was present in the T25 and T18 fusions, we cannot rule out the possibility that interacting proteins are localized in the periplasm of *E. coli*. However, based on the fact that the bacterial two-hybrid system allows the detection of protein interactions that occur either in the cytoplasm or at the inner-membrane level [7], this possibility is very unlikely.

Kocincova et al. have shown that the Erp protein is anchored to the surface of the bacterium by a carboxy-terminal hydrophobic domain and that it is easily released into the supernatant fraction [10]. From these data these authors proposed that Erp uses the carboxy-terminal domain to interact with some other molecules of the cell wall to achieve its correct structure. This finding led us to think that Rv1417 and Rv2617c might have the potential to anchor Erp by interaction with its carboxy-terminal domain. Nevertheless, here we demonstrated that this domain does not contribute to the interactions, thus suggesting that the membrane proteins Rv1417 and Rv2617c are not involved in the attachment of Erp to the cell surface. Remarkably, it has been demonstrated that the carboxy-terminal region of Erp is not essential for restoring the virulence and tissue damage of an *erp*-mutant strain of *M. tuberculosis *[17]. Therefore, based on the facts that this carboxy-terminal region, which is conserved through *Mycobacterium *species, is not implicated in protein interaction or virulence, one may speculate that the virulence properties of Erp are related with its capability of interaction. We found that the region involved in the interaction with Rv1417 and Rv2617c was located in the amino-terminal and central domains of Erp. However, we do not know whether the interactive domains are mapped at the central repetitive region and the amino-terminal region is relevant for protein folding, contributing to the protein association, or whether additional binding domains are present at the amino-terminal region.

The interaction of Rv1417 and Rv2617c with the repeat central domain of Erp is intriguing because this domain appears to be associated with a role in virulence and tissue damage of *M. tuberculosis *in mice. Recently, de Mendoça -Lima et al. [18] have shown that at early time-points of infection in lungs, Erp from *M*. *smegmatis *is not able to restore the wild type virulence of the *erp*-deficient *M. tuberculosis *strain, whereas Erp from pathogenic *M. leprae *induces a hypervirulent phenotype. In this work, we demonstrated that Erp from *M. leprae *(ML0091) was not able to interact either with Rv1417 or with Rv2617c, and that Erp from *M. smegmatis *(MSMEG6405) was able to interact with Rv1417 but not with Rv2617c. The absence of Rv2617c in species that do not belong to the MTC and the inability of both ML0091 and MSMEG6405 to interact with Rv2617c indicate that the Rv2617c-Erp interaction is restricted to species from the MTC. These interactions may have been acquired during divergent evolution when tuberculous *Mycobacterium *species arose. Thus, this work provides evidence suggesting multiple roles for members of the Erp family, which could explain why, being relevant for intracellular living, this proteins are present in saprophytic *Mycobacterium *species. On the other hand, the interaction of Rv1417 with the Erp member from *M. smegmatis *but not with the one from *M. leprae *suggests that the virulence role played by Erp orthologues is not related with their capacities of interaction with Rv1417. The investigation of such interactions in a variety of mycobacterial species will help to clarify this point.

Although the pairwise interactions of Rv1417, Rv2617c and Erp were clearly demonstrated, they may reflect a transient contact in an assembly pathway or stable interactions in a completed structure. In order to define this point, immunolocalization of the three proteins in the bacterial cell was investigated. In agreement with previous findings [1-3], Erp was localized mostly in culture supernatants, but also associated to the cell wall, whereas Rv1417 and Rv2617c were restricted to the cell membrane. This last finding is in agreement with the prediction of transmembrane helices in both Rv1417 and Rv2617c as determined by SOSUI and TMHMM software. An initial scan with InterProScan predicted a signal-peptide domain in these proteins that could not be confirmed neither by the specialized SOSUI nor by the SignalP programs. This is not unexpected since mycobacterial exported proteins without predicted signal sequences have been previously described [19]. Therefore, we postulate that Rv1417 and Rv2617c form a multimeric structure on the membrane which is, in turn, transiently associated to the Erp protein before being translocated to the cell envelope and the extracellular compartment. Although the experiments carried out in this study did not allow us to define whether Rv1417 and Rv2617c are localized in the cytoplasmic membrane or in the outer membrane, the former localization appears more plausible since these proteins are not predicted to be present in the outer membrane [20].

Our work shows that both Rv1417 and Rv2617c genes are transcribed in *in vitro *cultured *M. tuberculosis *and that *Rv1417 *is co-transcribed with the *RibH *gene, which is part of a riboflavin operon. We also found that *Rv1417 *orthologues are located in genomic regions that encode proteins that participate in the riboflavin metabolism in several species of *Actinomycetales*. Riboflavin (vitamin B2) is the precursor of the coenzymes flavin mononucleotide phosphate and flavin adenine dinucleotide phosphate, essential compounds for basic metabolism. It has been demonstrated that riboflavin biosynthesis is essential for *in vivo *survival of a number of bacterial species because of the scarcity of riboflavin in mammalian cells [21,22]. Although these findings strongly suggest a role of Rv1417 in riboflavin synthesis, more research is necessary to understand the association of riboflavin metabolism with the function of Erp and Rv2617c in *M. tuberculosis*, as well as to elucidate the biological significance of the protein interactions discovered in this study.

## Conclusion

We identified interactive partners of Erp, an *M. tuberculosis *protein involved in virulence, which will be the focus of future investigation to decipher the function of the Erp family protein.

## Methods

### Bacterial strains and culture media

All cloning steps were performed in *E. coli *DH5α, and *E. coli *BL21(DE3) was used for recombinant protein production. Complementation assays were carried out with the *E. coli *BTH101 strain (F^- ^*cya*-99, *ara*D139, *gal*E15, *gal*K16, *rps*L1 (Str^r^), *hsd*R2, *mcr*A1, *mcr*B1). *E. coli *strains were grown either in Luria-Bertani (LB) broth or on LB agar. Screening for the ability to ferment sugars was performed on M63 plates supplemented with 0.3% lactose. When necessary, ampicillin at 100 μg/ml and kanamycin at 50 μg/ml were added to the media. *Mycobacterium *strains were grown in either Middlebrook 7H9 medium supplemented with 0.05% Tween 80 or Middlebrook 7H11 medium, both supplemented with ADC (albumin -1 0.5%, dextrose 0.4%), and 0.5% glycerol. When necessary, 20 μg kanamycin ml^-1 ^was added to the media. Electrocompetent *M. tuberculosis *and *M. smegmatis *cells were prepared and transformed by electroporation, as described by Parish and Stoker [23].

### Construction of an *M. tuberculosis *library in a pUT18c plasmid

*M. tuberculosis *chromosomal DNA was partially digested with *AluI*. Then, 0.5- to 1-kbp purified DNA fragments were ligated to pUT18c plasmids digested with *Sma I*. The ligation mixture was electroporated into *E. coli *DH5α strain. Transformants were suspended in LB medium containing ampicillin, and plasmid DNA from this library was prepared. Analysis of randomly selected plasmids from individual clones showed that the average size of inserts was 0.5–1 kbp (data not shown). Plasmids were purified from pooled clones to generate a plasmid library.

### Construction of bait and prey plasmids

The entire open reading frames (ORFs) of Erp, P27, Rv1417 and Rv2617c (of *M. tuberculosis*) as well as the ORF of ML0091 (of *M. leprae*) and MSMEG6405 (of *M. smegmatis*) were PCR-amplified from their corresponding genomes and cloned as fusion to the T25 subunit of the adenylate cyclase of *Bordetella pertussis *into the bait vector pKT25 [7]. DNA fragments encoding for the amino-terminal region, the central domain, the carboxy-terminal region, the full-length Erp protein, and a version of *erp *deleted in the sequence encoding the carboxy-terminal region were PCR-amplified from *M. tuberculosis *chromosome and amplicons were cloned as fusions to the T18 subunit of the adenylate cyclase of *B. pertussis *into the prey vector pUT18C [7]. Details of primers used and plasmids generated are depicted in table 1.

**Table 1:** Plasmids and primers used in this study

Plasmid **	Primers	Sequence 5' – 3'^a^	Origin
**T25-Erp**	**Erpbaitup**	**ctgcaggggtgccgaaccgacgccga**	**This study**
	**ERPbaitlow**	**ggtaccttaggcgaccggcacggt**	

T25-1417	1417pktup*	ggatcccgtgaccgccgcaccgaac	This study
	1417pktlow*	ggtacctcagcgggcgcacaggtc	

**T25-2617**	2617pktup*	**ctgcagggatgagcatcagaccaacg**	**This study**
	2617pktlow*	**ggtaccttaaggccgcccgatgcc**	

T25-MlErp	ERPlepraeup	ggatccagtgccgaaccgacgccgatg	This study
	ERPlepraelow	ggatccctacgtgacaggaatcagtg	

**T25-MsErp**	**ERPsmgupok**	**ggtacctgtgccgaaccgccgtcga**	**This study**
	**ERPsmglow**	**ggtacctcagggtcgccgcgat**	

T18- ErpA	ERPputup	ctgcaggggtgccgaaccgacgccga	This study
	ERPaminochicolow	ggtaccctaggtcaggctgggcaccgg	

**T18-PGLTS**	**ERPPGLTSUp**	**ctgcagggggagcggcgatgccagcac**	**This study**
	**ERPPGLTSlow**	**ggtaccctattcgttggcgccgcccag**	

T18-ErpC	ERPCup	ctgcaggatcccgattacgacgccggt	This study
	ERPputlow	ggtaccttaggcgaccggcacggt	

**T18-Erp**	**ERPputup**	**ctgcaggggtgccgaaccgacgccga**	**This study**
	**ERPputlow**	**ggtaccttaggcgaccggcacggt**	

T18-ErpΔC	ERPputup	ctgcaggggtgccgaaccgacgccga	This study
	ERP A low	ggtaccctattcgttggcgccgcccag	

**pmip12-1417-myc**	**up1417myc**	**ggtaccgtgaccgccgcaccga**	**This study**
	**LowMyc**	**ggtaccttagtgatggtgatggtg**	

pmip12-2617c-myc	up2617cmyc	ggtaccatgagcatcagaccaac	This study
	LowMyc	ggtaccttagtgatggtgatggtg	

**pmip12**	-	-	[26]

ImpactVector 1.1	-	-	Wageninger UR

-	LeftOP1417***	**cagctggcacggaaagat**	**This study**
	RightOP1417***	**acgacacaccgatcacttca**	

** Plasmids contain the sequence obtained by amplification with the pair of primers on the right column.

* Primers used in PCR or RT-PCR analysis.

*** Primers used only in RT-PCR analysis.

^a^Restriction enzyme sites are underlined.

### Identification of gene products interacting with Erp by a bacterial two-hybrid assay

About 1 μg of plasmid DNA library was used to co-transform *E. coli *BTH101 competent cells harbouring the T25-Erp bait plasmid. Co-transformants were selected on M63 plates containing 0.3% lactose, ampicillin, kanamycin and 40 μg/ml of X-gal (5-bromo-4-chloro-3-indolyl-b-D-galactopyranoside). A small aliquot of the transformants was plated on rich LB medium containing ampicillin and kanamycin to select the presence of pUT18C derivatives. The total number of transformants deduced from these experiments demonstrated high efficiency of transformation of *E. coli *cells (data not shown). Blue colonies on M63 medium appearing after 5 days of incubation were assumed to contain pUT18C derivatives coding for potential proteins that interact with Erp. The pUT18C derivatives were isolated from these clones and reintroduced either into BTH101 cells containing T25-Erp or into BTH101 cells containing the empty pKT25 vector (as a negative control) to confirm the interactions and to exclude false positives. Hybrid plasmids from positive clones were sequenced, and genes coding for putative interactors were identified by BLAST searches in the *M. tuberculosis *genome database [8].

### Protein-protein interaction assays

*E. coli *BTH101 competent cells (which had a level of competency of 1 × 10^7^) were co-6 transformed with bait and prey plasmids (1 μg of each plasmid) and 1 × 10^6 ^cells were plated on M63 plates containing lactose, ampicillin, kanamycin and X-gal. The strength of interaction was assayed by counting the number of colonies on M63 plates.

In addition, controls of the efficiency of transformation were performed by plating 1 × 10^6 ^cells from each transformation mix on LB medium containing ampicillin and kanamycin. The number of colonies grown in the rich medium was approximately the same for all the reactions.

The interaction of fusion proteins encoded in T18-Zip and T25-Zip plasmids was used as a positive control.

### Statistical analysis

Means were tested for differences with Student's t test. Values were determined to be statistically significant at P < 0.05.

### Computer analyses

All identified ORFs were subjected to bioinformatic analysis including similarity searches, protein domain determination and genomic structure. Sequence similarity searches were performed by BLASTP [15]. The InterProScan software was used to search for conserved domains in the proteins against the InterPro database [11]. Transmembrane helix predictions were performed using the TMHMM Server [14]. Comparative genomic analysis was carried out with the String software [24] and BLAST analysis of the genome sequences.

### RT-PCR

RT-PCR reactions were performed from DNA-free RNA (1 μg) extracted from middle logarithmic-phase cultures of *M. tuberculosis *H37Rv as described previously [25]. The primers used in each assay are summarized in Table 1.

### Protein localization

Since attempts to raise antibodies against Rv1417 and Rv2617c were unsuccessful, the myc tag sequence was fused to the 3' end of both Rv1417 and Rv2617c and an anti-myc monoclonal antibody was used to recognise the recombinant protein in the recombinant mycobacterial strains. The full-length sequences of *Rv1417 *and *Rv2617c *were cloned into the ImpactVector 1.1-tag plasmid (Wageninger UR). The myc-tagged genes were PCR-amplified from the resulting plasmids and cloned in pmip12 [26] (see Table 1). Recombinant plasmids were used to transform *M. tuberculosis *H37Rv and *M. smegmatis *competent cells as described above. Subcellular fractioning of *Mycobacterium *strains was performed as described previously [9].

### Western blots

Western blot assays were performed as described previously [27] with the following antibodies: anti-P36/Erp Mab (1:500) [5] and anti-myc monoclonal antibody (1:100/Sigma-Aldrich). Alkaline phosphatase-conjugated anti-mouse immunoglobulin G (1:2000/Sigma-Aldrich) was used to detect anti-myc and anti-P36/Erp antibodies.

## Authors' contributions

LIK carried out the molecular biology and protein studies and participated in the draft of the manuscript. FCB and MVB carried out the RT-PCR assays and pull-down experiments, respectively. MS performed the informatics analysis. MPS helped in the elaboration of the manuscript. AAC and FB conceived the study and participated in its design and coordination and drafted the manuscript. All authors read and approved the final manuscript.

## Supplementary Material

Additional file 1 **In vivo interaction of Erp, Rv1417 and Rv2617c.** The data provided shows the *in vivo *interaction between Erp- Rv1417, Erp- Rv2617c and Rv1417- Rv2617c using the bacterial two- hybrid assay. Click here for file

Additional file 2 **In vitro interaction of Erp with either Rv1417 or Rv2617c by pull down assay.** The data provided shows the *in vitro *interaction between Erp- Rv1417 and Erp- Rv2617c using the GST- pull down assay. Click here for file

Additional file 3 **In vivo interaction of Rv1417 and Rv2617c.** The data provided shows the ability of Rv1417 and Rv2617c to form homodimers using the bacterial two hybrid assay. Click here for file
