# Supplementary material for: Identification of two proteins that interact with the Erp virulence factor from Mycobacterium tuberculosis by using the bacterial two-hybrid system
Source: BMC Mol Biol. 2009 Jan 21;10:3. doi: 10.1186/1471-2199-10-3 (PMC2639381; doi:10.1186/1471-2199-10-3)
Supplement: Additional file 2 — In vitro interaction of Erp with either Rv1417 or Rv2617c by pull down assay. The data provided shows the in vitro interaction between Erp- Rv1417 and Erp- Rv2617c using the GST- pull down assay. [file 1471-2199-10-3-S2.doc]

**
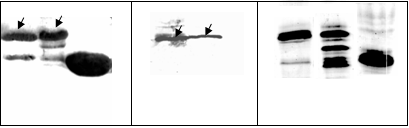
**

**1 2 3 1 2 3 1 2 3**

**KDa**

**40**

**26**

**A**

**BA**

**CA**

**Figure Additional file 2. *In vitro* interaction of Erp with either Rv1417 or Rv2617c by pull down assay:**

Glutathione S-transferase (GST) pull-down assays were used to confirm the interactions. GST-Rv1417 (lane 1), GST-Rv2617c (lane 2) and GST (lane 3) fusion proteins were immobilized on glutathione-Sepharose 4B beads (Amersham Bioscience) and incubated with recombinant Erp. The captured protein complexes were subjected to Western blot analyses either with anti-GST (Amersham Bioscience) at a 1:500 dilution (A) or with anti-P36/Erp Mab [1] at a 1:1000 dilution (B). (C) Analysis by SDS-PAGE of proteins bound on the glutathione-Sepharose 4B beads. Arrows indicate the interacting proteins.

The figure shows that both GST-1417 and GST-2617c fusions tagged to Glutathione Sepharose are able to bind Erp. No binding of Erp to GST (negative control) was detected.

The anti-GST antibody also revealed products of degradation of GST-fusion proteins.

**Materials and methods: pull-down assays**

The sequences encoding Rv1417 and Rv2617c were PCR-amplified from *M. tuberculosis* H37Rv, and cloned into pGEX-6P-1 (Amersham Bioscience). The resulting plasmids together with plasmid pMBA123, which contained the *erp* full length sequence under the *Plac* promoter [2], were introduced into *E. coli* BL21(DE3). Over-expression of recombinant proteins was induced with 1mM IPTG for 3 h. Cells were harvested and resuspended in 5 ml binding buffer (Tris-HCl 25mM, pH 7.5; 50 mM NaCl; Triton 1%) per gram of cell pellet. Soluble cell extracts from the cultures were prepared using a Fastprep FP120 bead-beater (Savant) for 20 s at a speed of 6.0 m s^-1^ with glass beads of 150-212 microns (Sigma-Aldrich). The lysates were clarified by centrifugation at 10000*g* for 15 min.

GST, GST-Rv1417 and GST-Rv2617c were bound to Glutathione–Sepharose beads (Amersham Biosciences) as described by the manufacturer. Recombinant proteins conjugated to Glutathione–Sepharose beads were separately incubated with non-purified Erp in binding buffer for 1 h at room temperature. After incubation, beads were washed three times with binding buffer and boiled with Laemmli sample buffer. Proteins were then resolved in 10% SDS–polyacrylamide gel electrophoresis and transferred to nitrocellulose membranes for Western blot analysis as described below.

**References**

1. Bigi F, Gioffre A, Klepp L, Santangelo MP, Velicovsky CA, Giambartolomei GH, Fossati CA, Romano MI, Mendum T, McFadden JJ, Cataldi A: **Mutation in the P36 gene of *Mycobacterium bovis* provokes attenuation of the bacillus in a mouse model.** *Tuberculosis* 2005, **85**: 221-226.

2. Bigi F, Alito A, Fisanotti JC, Romano MI, Cataldi A: **Characterization of a novel *Mycobacterium bovis* secreted antigen containing PGLTS repeats.** *Infect Immun* 1995, **63**: 2581-2586.
