# Supplementary material for: Identification of two proteins that interact with the Erp virulence factor from Mycobacterium tuberculosis by using the bacterial two-hybrid system
Source: BMC Mol Biol. 2009 Jan 21;10:3. doi: 10.1186/1471-2199-10-3 (PMC2639381; doi:10.1186/1471-2199-10-3)
Supplement: Additional file 3 — In vivo interaction of Rv1417 and Rv2617c. The data provided shows the ability of Rv1417 and Rv2617c to form homodimers using the bacterial two hybrid assay. [file 1471-2199-10-3-S3.doc]

**Figure Additional file 3. *In vivo* interaction of Rv1417 and Rv2617c**

*E. coli* BTH 101 cells were transformed with the plasmids described in the figure. For determinations of β-galactosidase activity, 1 ml of recombinant *E*. *coli* cultures were pelleted and measurements were performed following the procedure described by Miller [1]. The bars represent β-galactosidase activity expressed as Miller units [*A*420x1000/reaction time (min)x*A*600] ± S.D. of triplicate. *Significantly different (P < 0.05) from value of negative controls as calculated by Student’s t test.

Plasmids T18-1417 and T25-1417 are respectively pUT18c and pKT25 vectors carrying *Rv1417*.

Plasmids T18-2617 and T25-2617 are respectively pUT18c and pKT25 vectors carrying *Rv2617c*.

**References**

[1] Miller J: **Experiments in Molecular Genetics***.* Edited by J. H. Miller. Cold Spring Harbor, NY; 1972.
